# Developmental Defects of the Thyroid Gland: Relationship with Advanced Maternal Age

**DOI:** 10.4274/Jcrpe.560

**Published:** 2012-06-09

**Authors:** Heves Kırmızıbekmez, Ayla Güven, Metin Yıldız, Ayşe Nurcan Cebeci, Fatma Dursun

**Affiliations:** 1 Göztepe Education and Training Hospital, Department of Pediatric Endocrinology, İstanbul, Turkey; +90 216 566 40 00heveskirmizibekmez@yahoo.com

**Keywords:** Permanent congenital hypothyroidism, thyroid dysgenesis, hypoplasia, maternal age

## Abstract

**Objective:** Developmental defects of the thyroid gland are the most frequent causes of permanent congenital hypothyroidism. This study aimed to investigate the epidemiological features of patients with thyroid dysgenesis (TD).

**Methods:** Medical records of 234 patients with TD followed between the years 2008 and 2010 were evaluated retrospectively. Diagnosis was made by ultrasonography.

**Results:** Of 234 patients, 120 (51.3%) were male and 114 (48.7%) were female. Male to female ratio was 1.08 and there were no significant differences in epidemiologic and clinical findings between girls and boys. One hundred eighty-three patients (78.2%) were diagnosed as hypoplasia, 35 (14.9%) as thyroid agenesis, 4 as ectopic thyroid gland and 12 as hemiagenesis. The mean maternal age of the group was 28.9±0.4 years (range 18 to 45 years), which is significantly higher than the recently reported mean maternal ages for Turkish women.

**Conclusions:** Advanced maternal age was more prevalent in patients with TD. Our clinical and epidemiologic findings suggested no evidence of sexual dimorphism.

**Conflict of interest:**None declared.

## INTRODUCTION

Developmental defects of the thyroid gland, including aplasia (absent gland), hypoplasia (small gland) and ectopia (unusual location) are collectively referred to as thyroid dysgenesis (TD) (1). In iodine sufficient countries, 85% of permanent congenital hypothyroidism (CH) is due to TD (2). Most cases of TD are sporadic and their pathogenesis remains unknown (3).

Defects in embryogenesis, migration, differentiation or growth of the thyroid gland during organogenesis may lead to CH (4). Most CH patients with TD have a defect in thyroid migration resulting in ectopic thyroid tissue located in the lingual, suprahyoid or infrahyoid regions (4,5). The ectopic gland is often asymptomatic but may cause local symptoms (6). The second most common variant of TD is absence of detectable thyroid follicular cells, a condition commonly called agenesis. Developmental failure of this type may affect only one lobe of the gland, resulting in hemiagenesis, with or without an isthmus involvement.

Predominance of TD in girls has been declared in several studies (7,8). The prevalence is higher in Hispanics and Caucasians. Mutations associated with TD (TSHR, PAX8, NKX2-1, FOXE1, NKX2-5 and PAX9) account for only 2% of all cases (1,2,9). The possible role of humoral and environmental factors or post-zygotic events in thyroid development has not been excluded (10).

The aim of this study was to determine the epidemiological features of patients with TD and to investigate the possible reasons that could lead to defects in the development of the thyroid gland.

## METHODS

We retrospectively evaluated the medical records of 234 patients with TD (120 female and 114 male; mean age: 13.8±1.9 months) followed between 2008 and 2010 in Istanbul Göztepe Training Hospital at the Turkish Ministry of Health. The patient records contained information on age at referral, gender, birth weight, parental age, family history, clinical presentation, etiological diagnosis, hormonal levels, thyroid volumes and dose of levothyroxine (LT4) used as treatment. The diagnosis of CH was based on presence of high serum thyrotropin (TSH) with low serum free T4 (fT4) levels according to reference ranges.

All patients with CH had undergone an investigation for a developmental abnormality of the thyroid gland. Ultrasound assessment was made within two to three months of the diagnosis. A pediatric radiologist trained in the investigation of newborn babies and infants performed the assessment using special probes. Thyroid volume was estimated using the volumetric ellipsoid method (height x width x depth x correction factor) and a correction factor of 0.479, as recommended by the WHO (11). The length, width, and depth of each lobe were measured on the same workstation. Calculated thyroid volumes were assessed according to reference thyroid volumes in Turkish children (12). If thyroid gland was absent or imaged as a very tiny, imperceptible tissue, it was regarded as aplasia. Anti-thyroglobulin and anti-thyroid peroxidase antibodies were also studied in patients who presented at older ages in order to exclude autoimmune thyroiditis. Patients with autoimmune thyroiditis were not included in this study group even if they were shown to have TD.

Data obtained from the medical records were analyzed by SPSS 15.0 programme. Pearson’s correlation was used for the analysis of intercorrelations among the parameters investigated. Stepwise linear regression analyses were applied to determine the factors influencing thyroid volume. Comparison of mean maternal age with Turkish data was made by the student’s t-test. The chi-square test was used to investigate the distribution of maternal age at birth. A p-value of ≤0.05 was considered as statistically significant. Descriptive features were explored for differences between girls and boys by the independent-samples t-test. 

## RESULTS

Of 234 patients, 128 (54.7%) were referred to our hospital within their first two months of life by the national neonatal screening program group. Nineteen of the patients (8.1%) were infants hospitalized in our neonatal intensive care unit for different reasons and were diagnosed to have hypothyroidism. The remaining patients consisted of those who presented to our hospital with a variety of symptoms consistent with hypothyroidism and those in whom the diagnosis was based on incidentally detected laboratory abnormalities.The recorded prenatal complications were oligohydramnios in two mothers, gestational diabetes in one and preeclampsia in one. Birth weight was correlated with gestational age(r: 0.650, p<0.01). Twenty-seven (11.5%) of the patients were born prematurely and 18 (7.6%) were low birth weight infants. Thyroid volume was found to show a significant positive correlation with referral age (p<0.01).Parental consanguinity, which is known to be a risk factor for genetic disorders, was present in 69 patients (29.5%). The reported incidence of thyroid disease in the family was 35.5%.

Male to female ratio was 1.08. Distribution of epidemiologic findings and clinical features according to gender did not show any significant differences between male and female patients. These findings included family history for thyroid disease, parental consanguinity, etiology of thyroid gland disorder, birth weight, maternal and paternal ages.Parental ages at birth were recorded in 215 patients. These data showed that advanced parental age was more common in patients with TD. As expected, there was a correlation between maternal and paternal ages (r: 0.831, p<0.01). Maternal age data were compared with the findings of relevant studies on Turkish women, in which the mean maternal age was reported as 27 years (13,14). In our study group, 118 mothers (54%) were older than 27 years and maternal age ranged between 18 and 45 years. Mean maternal age of mothers of infants with TD (28.9±0.4) was found to be significantly higher as compared by the student’s t-test with the references cited above (p<0.05). Distribution of maternal ages according to age groups was also investigated. Only two mothers (0.9%) were younger than 18 years, 54 (25.1%) were between 19 and 24 years old, 69 (32%) were between 25 and 29, 47 (21.8%) between 30 and 34, and 43 (20%) were older than 35 years. These results were compared with the cited reference data (13) (Table 1).

In our study group, the most common developmental defect was hypoplasia of the thyroid gland. Thyroid gland volumes were below normal ranges for age in 183 patients (78.2%), with normal localization. Of 35 patients (14.9%) considered as thyroid aplasia, 19 patients had no detectable thyroid gland, while 16 were reported to have a very tiny gland with a calculated volume between 0.03 mL and 0.2 mL. An ectopic thyroid gland was present in 4 patients. Thyroid tissu detected in the anterior tracheal region in one of these patients and three had sublingually located thyroid glands. Hemiagenesis was detected in 12 patients - 7 with right- and 5 with left-lobe agenesis. In 5 of the 7 patients with right-lobe agenesis and in 4 of the 5 patients with left-lobe agenesis, the other lobe was hypoplastic. 

## DISCUSSION

Newborn screening programs implemented in several parts of the world have contributed to a better understanding of the pathogenesis of CH. The etiology is important in identifying the nature of the disorder (transient or permanent) and for better clinical management (15). Thyroid dysgenesis due to abnormal thyroid gland development is the most common cause of permanent CH (3). A heterogeneous group of developmental abnormalities, including thyroid hemiagenesis, ectopic thyroid tissue, cysts of the thyroglossal duct and thyroid hypoplasia, accounts for about 85% of all cases with CH (7).

The underlying etiology of most cases of TD is not well understood. Although most cases of TD are sporadic, the 2% familial occurrence, the higher prevalence of TD in babies of certain ethnic groups (e.g. Hispanics) than in others (e.g. African Americans) and the increased incidence in babies with Down syndrome suggest that genetic factors might play a role in some cases. The transcription factors PAX8, NKX2-1, FOXE1, NKX2-5 and PAX9 would seem to have an important role in thyroid organogenesis and/or migration. Potential etiologic mechanisms include epigenetic phenomena, early somatic mutations, or postzygotic stochastic events such as a low supply of blood, oxygen or other nutrients, which may alter or disrupt a genetic cascade of development of the thyroid at a crucial time (16).In the patients referred to our clinic with a diagnosis of hypothyroidism, ultrasonography for evaluating thyroid gland volume had been performed after the initiation of treatment. In CH, ultrasound may show no thyroid tissue in the neck because of either ectopia or agenesis, or alternatively, a small hypoplastic thyroid gland may be detected. However, thyroid scan is the “gold standard” to discriminate between dysgenesis and dyshormonogenesis cases, if performed before initiation of LT4 treatment, especially in infants with significantly elevated TSH levels. Ultrasound cannot replace scintigraphy in this regard, but in case of inadequacies, may be helpful for differential diagnosis and treatment predictions. Since most CH patients with TD have a defect in the migration of the thyroid gland, resulting in presence of ectopic thyroid tissue in a lingual, suprahyoid or infrahyoid location, identification with ultrasound is successful. As shown in recent studies, lingual thyroid, the most common ectopic localization, can be well identified by using midline sagittal and posterior coronal views of the mouth floor (17).

Thyroid ectopia is known to be the cause of approximately two-thirds of TD cases (2,18). Ectopic thyroid tissue may lead to CH, but such tissues are also discovered incidentally in some asymptomatic subjects, raising the possibility that many cases are never diagnosed (19). The absence of symptoms in most subjects suggests that the ectopic thyroid tissues may function normally (20).In our patient group with TD, we found an increased incidence of thyroid hypoplasia, rather than ectopia, a finding which is not in accordance with other reported TD series (21,22,23). This finding may be due to the fact that we did not perform thyroid scintigraphy in our patients. A potential candidate gene to explain the development of isolated thyroid hypoplasia is the TSH receptor. Because this gene is only expressed after the thyroid gland has migrated into the neck, loss-of-function mutations in this gene could only explain the finding of hypoplasia but not ectopy (16).

A higher prevalence of TD in girls, reported in previous publications, was not observed in our patients (18,23,24). Our study group, which consisted of 120 (51.3%) male and 114 (48.7%) female patients (M/F: 1.08), did not show any significant differences in clinical and epidemiological findings suggesting sexual dimorphism. Although the known mutations are present in only a small number of cases, parental consanguinity in 69 patients (29.5%) may be considered a finding suggesting that genetic factors may have had a role in the pathogenesis.

One remarkable finding in this study was the advanced maternal age in this series, as compared with recent data on maternal age in Turkish women. Maternal age has been investigated in a recent cross-sectional, epidemiologic study conducted on 502 women who gave birth in a large Obstetrics and Gynecology Hospital in Ankara (13). The mean maternal age at birth was found to be 27 years in this group. This figure is in agreement with the 2009 data of the Turkish Statistical Institute (14). Advanced maternal age is known to be associated with conditions such chromosome defects, hematologic malignancies, urinary tract abnormalities, high risk pregnancies and deliveries, but, to our knowledge, has not previously been reported in mothers of infants with TD. It is becoming increasingly evident that the clinical findings of TD may be associated with a multiplicity of etiologic mechanisms (25,26).

In conclusion, in this study designed to investigate the clinical and epidemiological features of TD, we have shown that advanced maternal age is more common in cases with TD. The role of humoral and environmental factors in TD is not well known, but advanced maternal age may increase the possibility of new mutations in genes encoding some transcription factors associated with thyroid gland development. 

## Figures and Tables

**Table 1:**
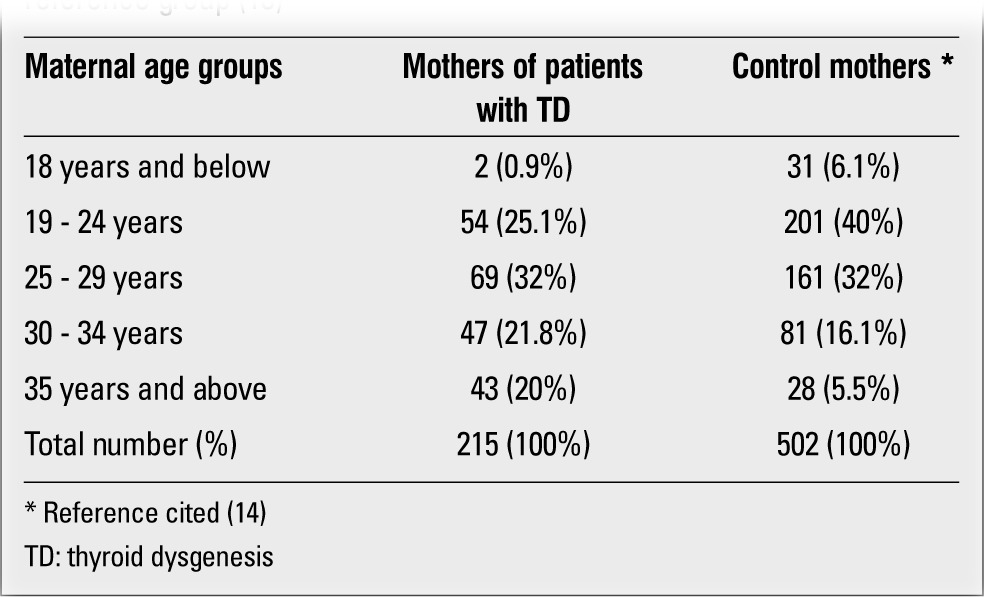
Maternal age distribution in the study group as compared to areference group (13)
